# Development and external validation of a decision-support model for predicting failed closed reduction in pediatric supracondylar humerus fractures: a multicenter retrospective study

**DOI:** 10.3389/fped.2026.1824240

**Published:** 2026-04-24

**Authors:** Rong Guo, Yi Zhou, Xiaoxuan Dai

**Affiliations:** 1 Department of Orthopedics, Xiangtan Central Hospital (Hunan University Affiliated Hospital), Xiangtan, Hunan, China; 2 Department of Trauma Surgery, Zhuzhou Central Hospital, Zhuzhou, Hunan, China

**Keywords:** closed reduction, open reduction, pediatric, prediction model, supracondylar humerus fracture

## Abstract

**Background:**

Closed reduction and percutaneous pinning (CRPP) is the standard treatment for pediatric supracondylar humerus fractures (SCHF). However, closed reduction can be challenging in a subset of patients and may fail, requiring conversion to open reduction. A practical preoperative tool to predict failed closed reduction remains lacking.

**Methods:**

This multicenter retrospective cohort study included pediatric patients with SCHF who underwent intended CRPP at Xiangtan Central Hospital and Zhuzhou Central Hospital between 2020 and 2025. The primary outcome was failed closed reduction, defined as intraoperative conversion to open reduction (including mini-open exposure) after attempted standard closed reduction maneuvers and before definitive K-wire fixation. Candidate predictors were selected using least absolute shrinkage and selection operator (LASSO) regression and incorporated into a multivariable logistic regression model. Model performance was assessed by discrimination, calibration, and decision curve analysis (DCA). External validation was performed using an independent cohort.

**Results:**

A total of 179 patients were included (development cohort, *n* = 86; validation cohort, *n* = 93). Conversion to open reduction occurred in 34.1% of patients. In the final multivariable model, displacement direction, preoperative Baumann angle, and the presence of a medial spike/entrapment sign were retained as predictors. The model demonstrated good discrimination in the development cohort (AUC = 0.842) and acceptable discrimination in the external validation cohort (AUC = 0.727). Calibration showed good agreement between predicted and observed risks, and decision curve analysis suggested potential clinical utility across relevant threshold probabilities.

**Conclusions:**

We developed and externally validated a practical decision-support model for predicting failed closed reduction (conversion to open reduction) in pediatric SCHF. This tool may facilitate preoperative planning and timely preparation for open reduction when needed, potentially improving operative efficiency and patient safety. However, given the retrospective design and the limited regional cohort, further prospective validation is required before routine clinical implementation.

## 1 Introduction

Pediatric supracondylar humerus fractures (SCHF) represent the most common elbow fractures in children and frequently require surgical management. Closed reduction and percutaneous pinning (CRPP) remains the preferred treatment owing to its minimally invasive nature and favorable outcomes (1, 2). In selected cases, adjunctive or alternative reduction/fixation strategies, such as joystick-assisted reduction, temporary external fixation constructs, or elastic stable intramedullary nailing, have also been described. However, closed reduction followed by K-wire fixation remains the standard treatment for most displaced pediatric supracondylar humerus fractures.

Despite standardized treatment protocols, successful closed reduction is not always achievable. Repeated reduction attempts may prolong operative duration and increase risks of soft tissue injury, neurovascular compromise, and conversion to open reduction (3). Early identification of fractures likely to present reduction difficulty could improve surgical planning and patient safety (4).

Previous studies have mainly focused on fixation techniques, complication profiles, or postoperative outcomes, whereas relatively few have specifically addressed preoperative prediction of failed closed reduction itself. In addition, most available evidence is derived from single-center experience and lacks externally validated, clinically interpretable tools that can be applied before surgery using routinely available radiographic findings (5).

Therefore, this multicenter study aimed to develop and externally validate a preoperative decision-support model to predict failed closed reduction (conversion to open reduction) in pediatric Gartland type III extension-type SCHF scheduled for operative treatment. By focusing on variables available before surgery, the model was intended to support preoperative risk stratification, surgical preparation, and caregiver counseling.

## 2 Materials and methods

### 2.1 Study design and participants

This multicenter retrospective cohort study was conducted at Xiangtan Central Hospital and Zhuzhou Central Hospital. Pediatric patients treated between 2020 and 2025 were screened.At both participating institutions, preoperative neurovascular assessment was routinely performed. Patients presenting with a pale/white pulseless extremity were considered unsuitable for repeated closed reduction attempts and were managed with urgent open exploration/reduction according to institutional practice; such cases were generally not included in the intended-CRPP cohort. In contrast, in pink pulseless cases, a cautious trial of closed reduction could be performed, usually limited to one or two attempts. If satisfactory alignment could not be achieved promptly, or if perfusion remained concerning after reduction, open reduction and/or vascular exploration was undertaken at the discretion of the treating surgeon.

#### 2.1.1 Inclusion criteria

age ≤14 years; 2) radiographically confirmed displaced extension-type supracondylar humerus fracture classified as Gartland type III and scheduled for operative treatment;3) intended treatment with CRPP; 4) available preoperative anteroposterior and lateral radiographs.

#### 2.1.2 Exclusion criteria

1) Open fractures; 2) Pathological fractures; 3) Previous ipsilateral elbow surgery; 4) Concomitant fractures requiring alternative fixation; 5) Missing key clinical or imaging data.This study was approved by the institutional ethics committees of both participating centers, and the requirement for informed consent was waived due to the retrospective design.

Accordingly, the present model was designed for patients in whom standard closed reduction was considered an appropriate initial strategy, rather than for children with obvious vascular compromise requiring immediate open exploration. Therefore, the model should be interpreted as applying primarily to surgically treated Gartland type III extension-type fractures in which standard closed reduction is initially intended, rather than to all supracondylar fractures or cases requiring immediate vascular exploration.

### 2.2 Cohort allocation

Patients from Xiangtan Central Hospital formed the development cohort, and those from Zhuzhou Central Hospital served as the external validation cohort.

### 2.3 Outcome definition

Failed closed reduction was defined as intraoperative conversion to open reduction (including mini-open exposure) after attempted standard closed reduction maneuvers and before definitive K-wire fixation, because an acceptable reduction could not be achieved or maintained. The outcome was ascertained from operative notes and procedure records.

### 2.4 Data collection

**Figure 1:**
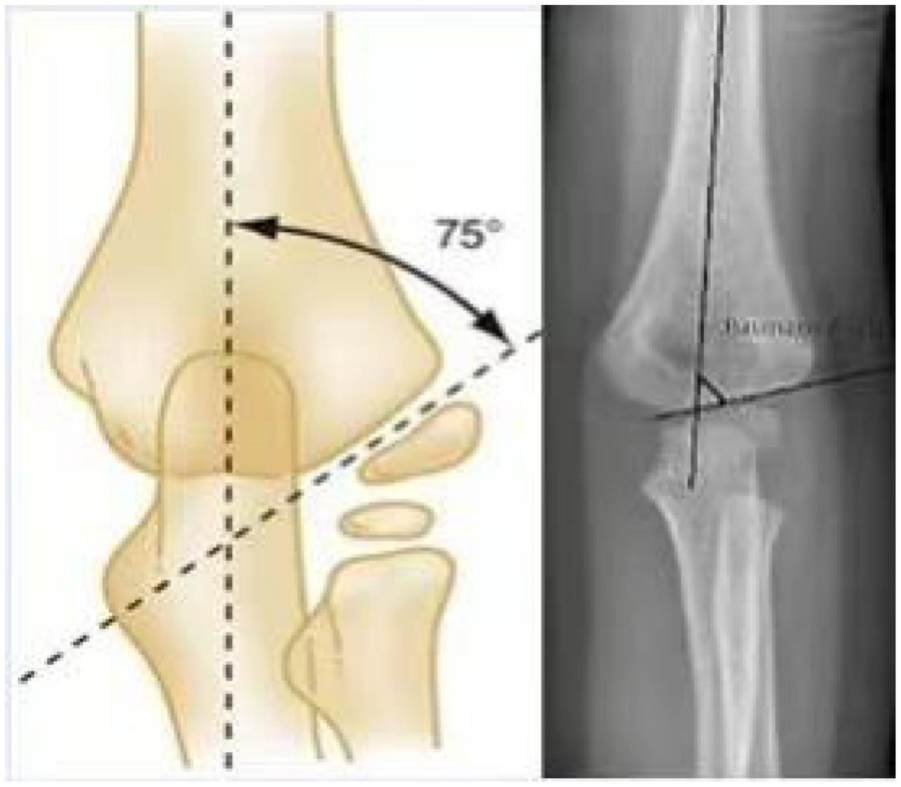
Baumann angle measurement schematic on AP radiograph.

Demographic characteristics, injury-related variables, neurovascular status, and radiographic parameters were extracted from electronic medical records.Radiographic measurements included:

1) **Displacement direction** was classified based on the direction of distal fragment displacement on the lateral/AP views as posteromedial or posterolateral (the most common pattern in extension-type SCHF); fractures not fitting these patterns were categorized as other.
2) **Preoperative Baumann angle (°)** was measured on the anteroposterior (AP) view as the angle between the long axis of the humeral shaft and the physeal line of the lateral condyle; higher values indicate greater coronal malalignment. (Figure 1)
3) **Rotational sign** was recorded as present when radiographs suggested rotational malalignment (e.g., asymmetric appearance/overlap of the distal fragment on AP view and/or incongruence of the distal fragment on lateral view), and absent otherwise.

Two independent observers performed all measurements using a standardized protocol; disagreements were resolved by consensus, although formal interobserver reliability analysis was not performed in this retrospective study.

### 2.5 Statistical analysis

Continuous variables are presented as mea*n* ± standard deviation or median (interquartile range), while categorical variables are expressed as frequencies and percentages. Patients with missing values in any candidate predictors were excluded (complete-case analysis). Predictor selection was performed using LASSO regression. Although rotational sign was retained by LASSO, it was not included in the final multivariable model because the negative category was rare in the development cohort, which could lead to unstable estimation in standard logistic regression.Variables with non-zero coefficients were entered into multivariable logistic regression analysis to construct the prediction model.Given the limited number of outcome events in the development cohort, the final multivariable model was restricted to a small number of clinically relevant predictors to reduce the risk of overfitting.Model performance was evaluated by the area under the ROC curve (AUC), calibration plots, Brier score, and decision curve analysis (DCA). Internal validation was conducted using bootstrap resampling. External validation was performed using the independent cohort from Zhuzhou Central Hospital. All analyses were performed using R software (version 4.3).

## 3 Results

**Figure 2:**
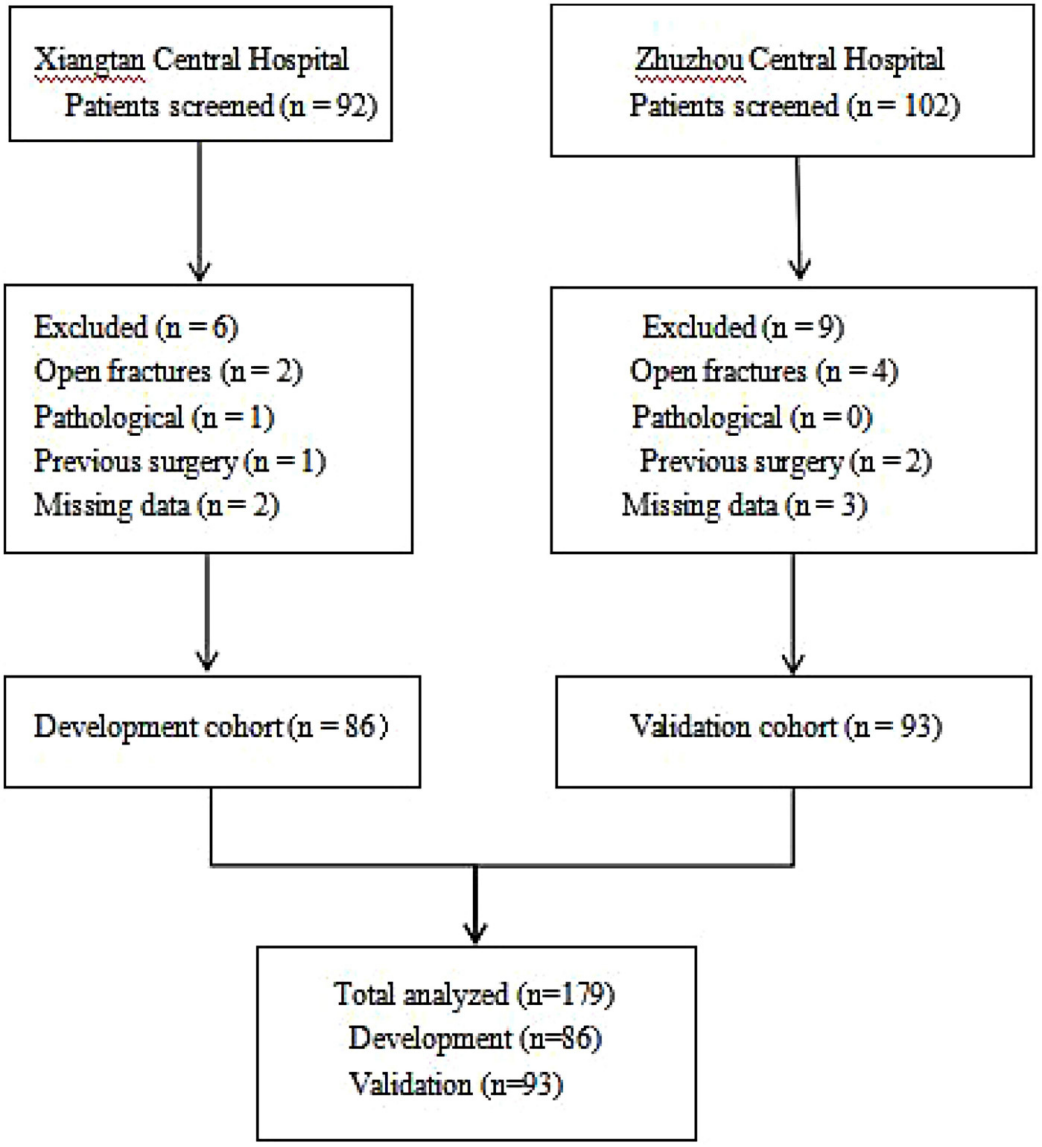
Flow diagram of patient selection and cohort allocation. Patients from Xiangtan Central Hospital constituted the development cohort, and those from Zhuzhou Central Hospital served as the external validation cohort.

A total of 194 patients were screened, and 179 met the inclusion criteria (Figure 2). The development cohort included 86 patients, and the external validation cohort comprised 93 patients.

Conversion to open reduction occurred in 61 patients (34.1%) overall, including 29 cases (33.7%) in the development cohort and 32 cases (34.4%) in the validation cohort; the conversion rate did not differ significantly between centers (Table 1).

**Table 1:** Baseline characteristics of the development and external validation cohorts.

Variable	Total (*n* = 179)	Development cohort (*n* = 86)	Validation cohort (*n* = 93)	*P* value
Age (years)	5.9 ± 2.4	5.9 ± 2.7	5.9 ± 2.0	0.973
Weight (kg)	22.2 ± 8.9	23.4 ± 10.2	21.1 ± 7.3	0.085
Time from injury to surgery (h)	21.6 ± 14.5	20.3 ± 17.8	22.7 ± 10.5	0.284
Preoperative Baumann angle (°)	77.6 ± 14.0	77.7 ± 19.0	77.4 ± 7.0	0.902
Number of reduction attempts	2.2 ± 0.8	1.9 ± 0.8	2.4 ± 0.7	<0.001
Reduction time (min)	22.3 ± 8.8	20.6 ± 8.7	23.8 ± 8.7	0.017
Sex				0.823
Male	117 (65.4%)	55 (64.0%)	62 (66.7%)	
Female	62 (34.6%)	31 (36.0%)	31 (33.3%)	
Injured side				0.689
Left	94 (52.5%)	47 (54.7%)	47 (50.5%)	
Right	85 (47.5%)	39 (45.3%)	46 (49.5%)	
Displacement direction				0.898
Type 1	29 (16.2%)	14 (16.3%)	15 (16.1%)	
Type 2	115 (64.2%)	54 (62.8%)	61 (65.6%)	
Type 3	35 (19.6%)	18 (20.9%)	17 (18.3%)	
Abnormal radial pulse	174 (97.2%)	81 (94.2%)	93 (100.0%)	0.024
Nerve injury	52 (29.1%)	26 (30.2%)	26 (28.0%)	0.865
Anterior humeral line crosses capitellum	28 (15.6%)	14 (16.3%)	14 (15.1%)	0.984
Rotational sign	172 (96.1%)	84 (97.7%)	88 (94.6%)	0.446
Medial spike/entrapment sign	113 (63.1%)	53 (61.6%)	60 (64.5%)	0.806
Conversion to open reduction	61 (34.1%)	29 (33.7%)	32 (34.4%)	1.000

**Figure 3:**
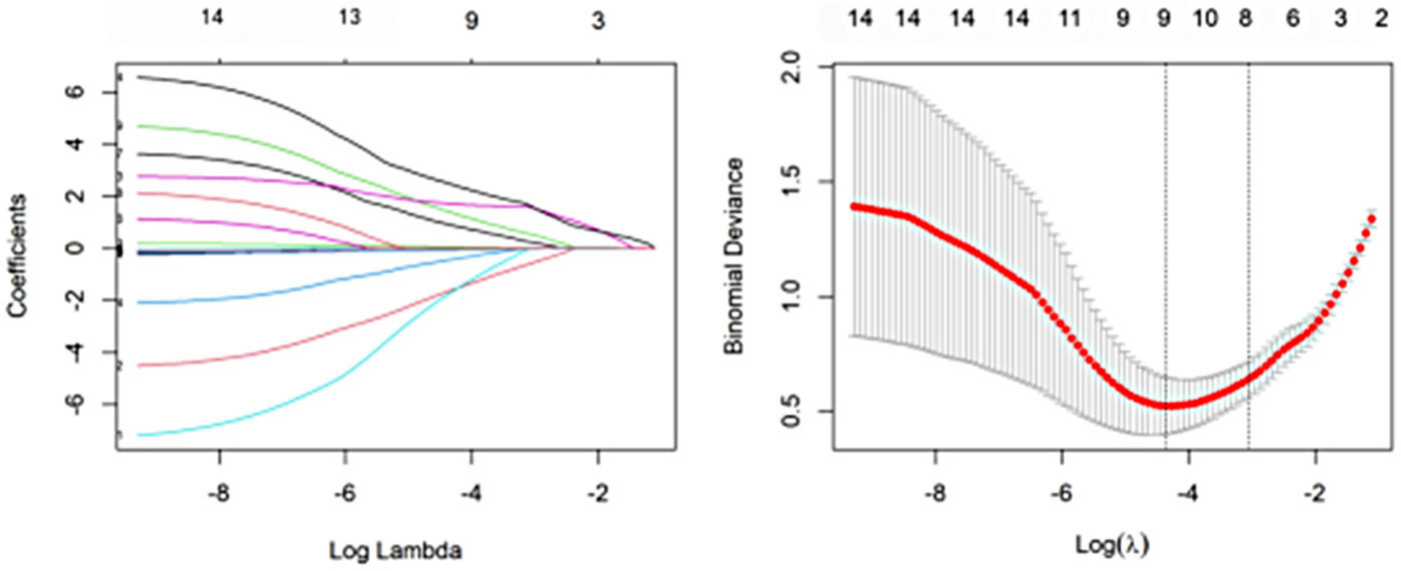
LASSO for predictor selection. **(A)** LASSO coefficient paths. **(B)** Ten-fold cross-validation to select the optimal penalty parameter (*λ*).

To reduce model overfitting and identify the most informative predictors, least absolute shrinkage and selection operator (LASSO) logistic regression was applied in the development cohort. A total of 14 candidate variables were entered into the LASSO model, and the optimal penalty parameter (*λ*) was determined by 10-fold cross-validation. four predictors with non-zero coefficients were retained: rotational sign, displacement direction, preoperative Baumann angle, and medial spike/entrapment sign (Figure 3). These variables were subsequently included in the multivariable logistic regression model to construct the final decision-support nomogram.

However, because the absence of rotational sign was rare in the development cohort, it was not included in the final multivariable model. In the final model, other/uncertain displacement was associated with higher odds of conversion to open reduction compared with posterolateral displacement (OR 10.579, 95% CI 1.272–87.954, *P* = 0.029), and the medial spike/entrapment sign showed a strong association with conversion (OR 37.813, 95% CI 6.681–214.015, *P* < 0.001); Baumann angle was not significantly associated with conversion (OR 0.987 per 1° increase, 95% CI 0.937–1.039, *P* = 0.617), with no substantial multicollinearity (all VIFs < 2.0) (Table 2).

**Table 2:** Multivariable logistic regression with VIF for conversion to open reduction (development cohort, *n* = 86).

Predictor	VIF	OR (95% CI)	*P* value
Displacement direction (overall)			0.069
Posteromedial vs Posterolateral (ref)	1.604	1.608 (0.350–7.385)	0.541
Other/uncertain vs Posterolateral (ref)	1.581	10.579 (1.272–87.954)	0.029
Preoperative Baumann angle (per 1° increase)	1.053	0.987 (0.937–1.039)	0.617
Medial spike/entrapment sign (present vs absent)	1.040	37.813 (6.681–214.015)	<0.001

**Figure 4:**
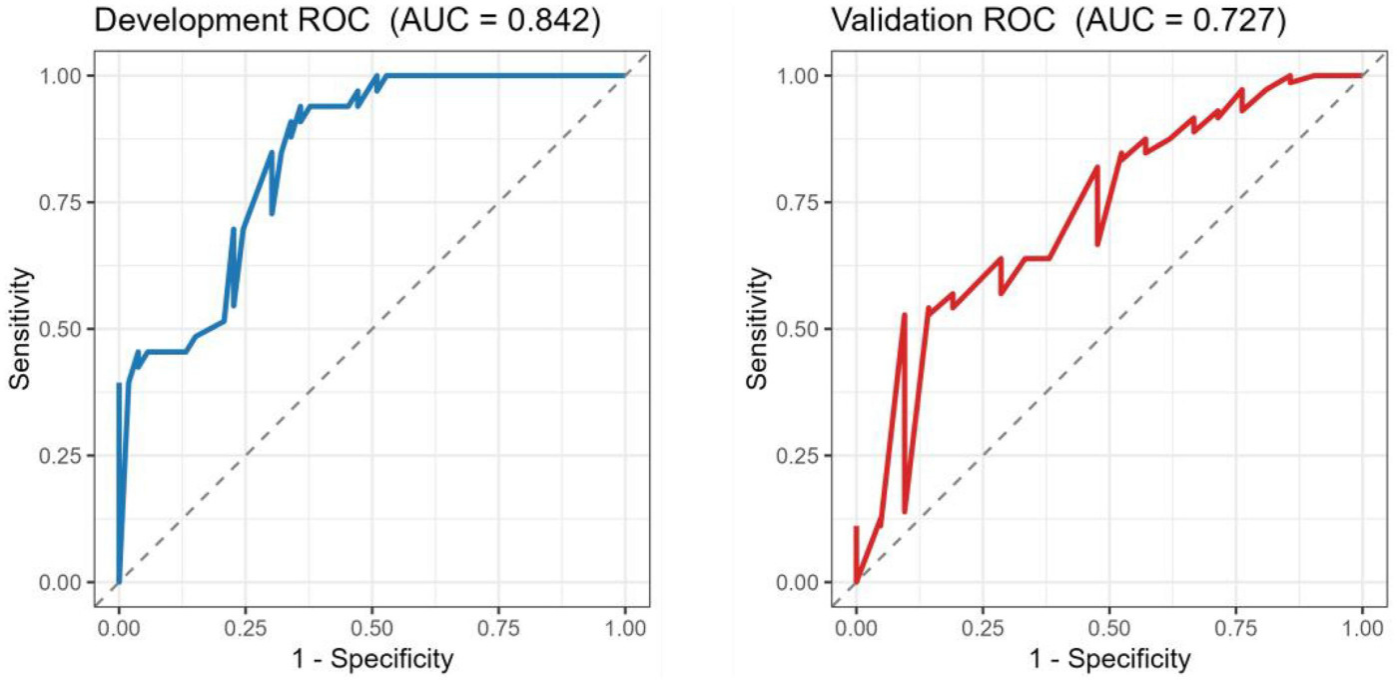
Receiver operating characteristic (ROC) curves of the final model in the development cohort **(A)** and external validation cohort **(B).**

The discriminative performance of the final model was evaluated using ROC analysis (Figure 4). The model demonstrated good discrimination in the development cohort with an AUC of 0.842, and maintained acceptable discrimination in the external validation cohort with an AUC of 0.727.

**Figure 5:**
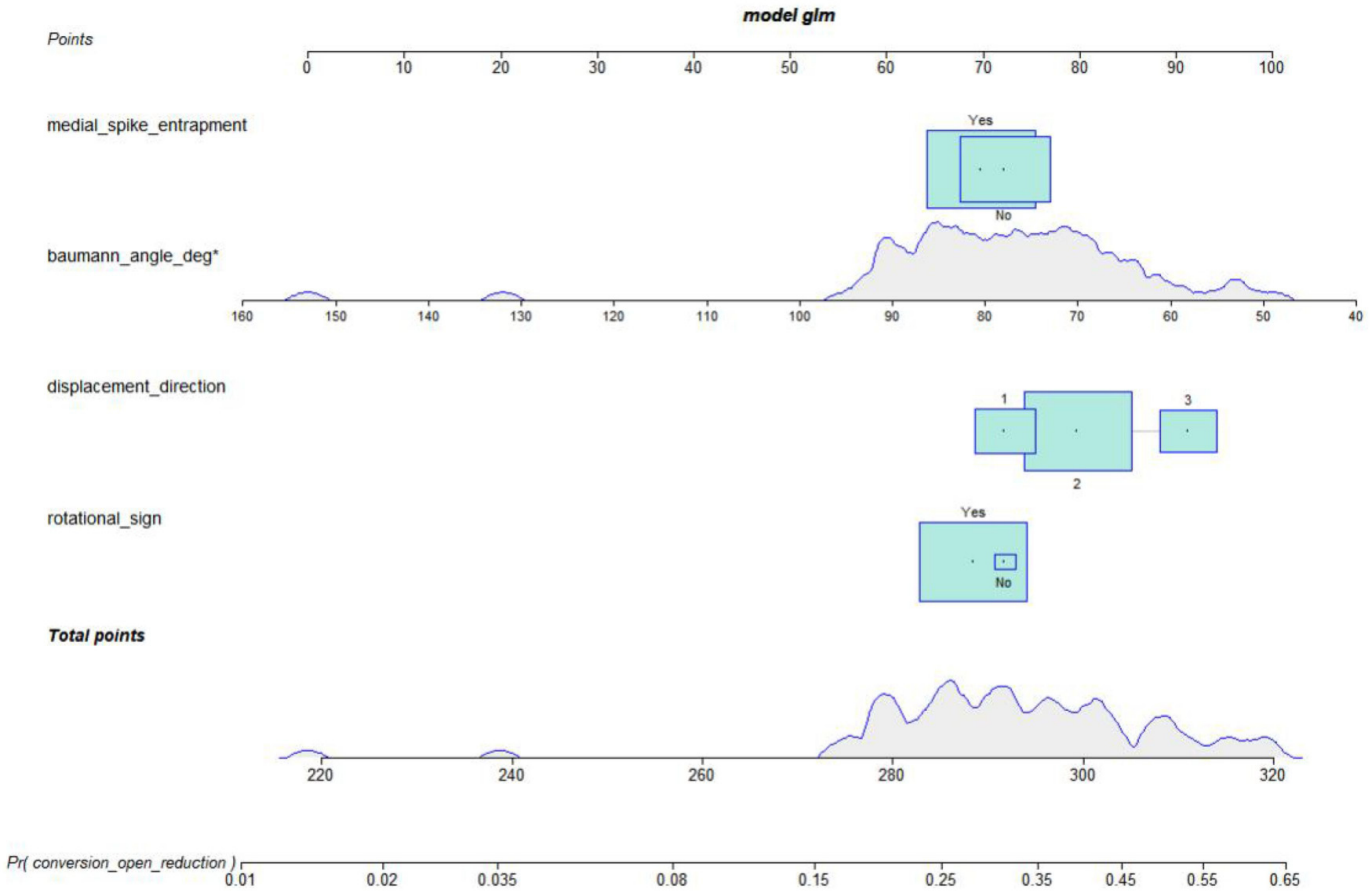
Nomogram of the final model for predicting conversion to open reduction.

Based on the final multivariable logistic regression model, a nomogram was constructed to provide an individualized estimate of the probability of conversion to open reduction (Figure 5).

**Figure 6:**
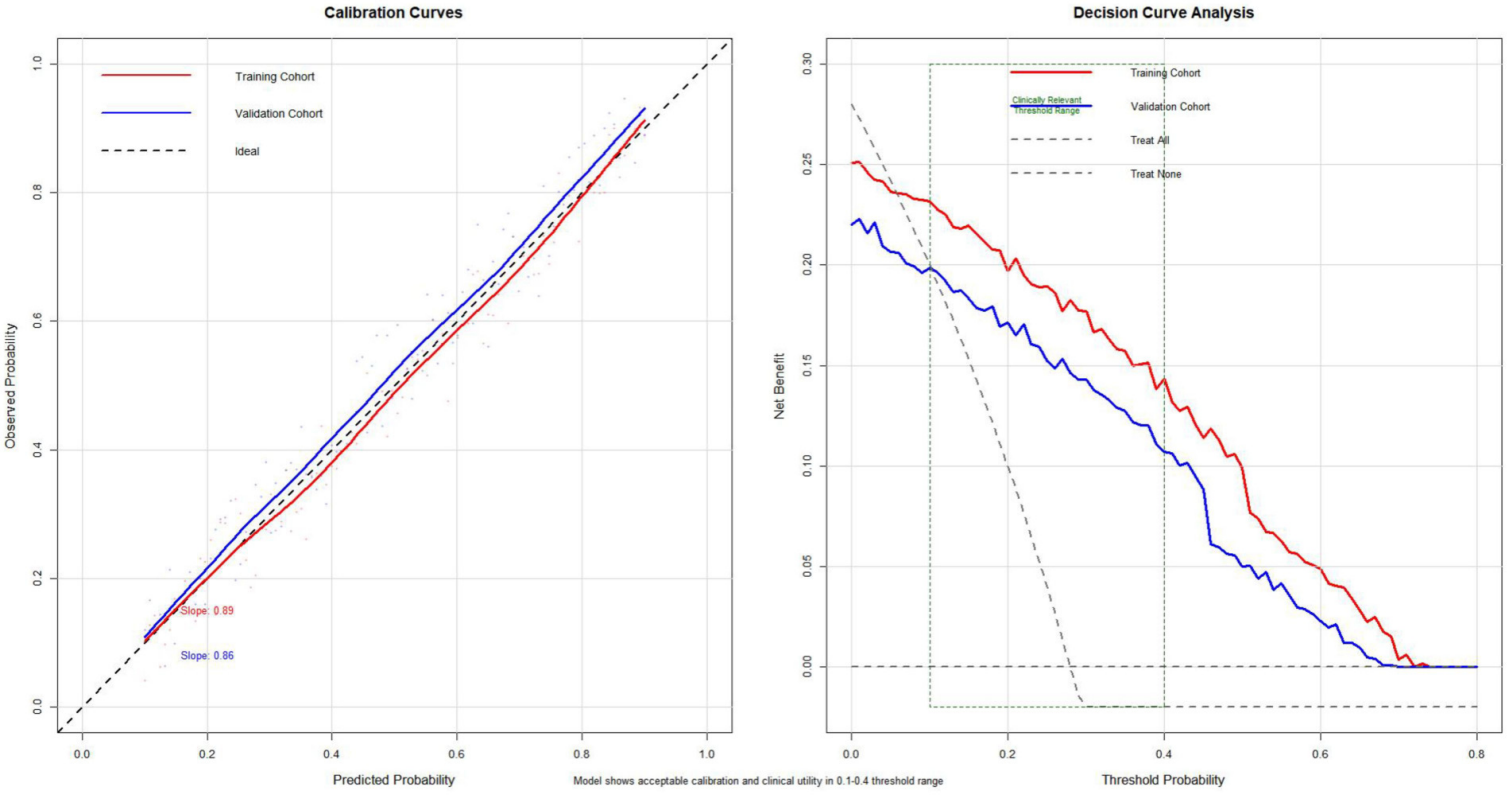
Calibration curves and decision curve analysis of the final model. (Left) Calibration plots for the development (red) and external validation (blue) cohorts; the dashed line represents ideal calibration. (Right) Decision curve analysis showing the net benefit of the model compared with “treat-all” and “treat-none” strategies across threshold probabilities; the model provides net clinical benefit within clinically relevant thresholds.

Calibration curves demonstrated good agreement between predicted and observed probabilities in both cohorts. The calibration slopes were 0.89 in the development cohort and 0.86 in the external validation cohort, indicating acceptable calibration with only mild deviation from the ideal line. Decision curve analysis suggested that applying the model would yield a higher net benefit than “treat-all” or “treat-none” strategies across clinically relevant threshold probabilities, particularly within the 0.10–0.40 range (Figure 6).

## 4 Discussion

Pediatric supracondylar humerus fractures (SCHF) are common injuries in children, and closed reduction with percutaneous pinning remains the standard treatment for displaced fractures (6). However, a subset of patients require conversion to open reduction when adequate reduction cannot be achieved by closed maneuvers, which may prolong operative time and increase soft-tissue trauma (7).

In this multicenter retrospective study, we developed and externally validated a pragmatic preoperative prediction model for failed closed reduction (conversion to open reduction). The model showed good discrimination in the development cohort (AUC = 0.842) and acceptable discrimination in the external validation cohort (AUC = 0.727), with acceptable calibration (slopes 0.89 and 0.86). Decision curve analysis suggested a net clinical benefit across clinically relevant threshold probabilities (approximately 0.10–0.40).Within this threshold range, use of the model may help identify patients for whom surgeons should be more prepared for possible conversion to open reduction.supporting potential utility in preoperative planning (8).

Among predictors, the medial spike/entrapment sign showed the strongest association with conversion, which is clinically plausible because irreducibility is often related to mechanical blockage by soft-tissue interposition or buttonholing (9). Our findings are consistent with emerging evidence that medial spike–related radiographic morphology correlates with the need for open reduction in SCHF (10, 11). Compared with prior work that focused mainly on treatment techniques and postoperative outcomes, our model emphasizes preoperative risk stratification using routinely available radiographic/clinical features and includes an independent external validation, which is essential for assessing transportability. However, some potentially relevant factors, such as soft-tissue interposition, surgeon experience, and more detailed fracture morphology, were not available in a standardized manner in this retrospective dataset.

Preoperative vascular status also deserves emphasis. From a practical standpoint, a pale/white pulseless extremity should raise strong concern for significant vascular compromise and may warrant urgent open exploration/reduction rather than repeated closed reduction attempts. By contrast, in a pink pulseless extremity, a limited and carefully performed trial of closed reduction may be reasonable, but repeated attempts should be avoided because they may delay definitive management and exacerbate soft-tissue injury. In this context, preoperative circulatory compromise is clinically relevant not only as a marker of injury severity but also as a factor that may lower the threshold for open reduction.

We also considered the potential relationship between time from injury to surgery and failed closed reduction. Although time from injury to surgery was recorded in our cohort, it was not retained as an independent predictor in the final model. This may suggest that its effect is less direct than that of radiographic indicators of fracture morphology. Nevertheless, delayed surgery could still contribute to swelling, muscle spasm, and soft-tissue interposition, which may increase reduction difficulty in selected cases. Larger prospective studies are needed to further clarify this association.

This tool may help surgeons anticipate difficult reductions, prepare for possible open exposure, optimize operative workflow, and facilitate informed discussions with caregivers. In practice, the model is intended to support preoperative planning rather than to serve as a stand-alone decision tool. Importantly, the lower AUC in external validation is expected when models are transported across settings, potentially reflecting differences in case mix, surgeon experience, and conversion thresholds (12).

Several limitations should be acknowledged. First, the retrospective design may have introduced selection bias and unmeasured confounding. Second, the model was developed for surgically treated Gartland type III extension-type fractures in which closed reduction was initially intended, and therefore may not be generalizable to all SCHF presentations. Third, predictor variables were limited to factors that could be reliably extracted from routine records and radiographs, while other potentially relevant factors, such as soft-tissue interposition, surgeon experience, and more detailed fracture characteristics, were not fully captured. Fourth, the sample size was relatively limited, and overfitting cannot be completely excluded despite model simplification and external validation. Finally, both centers were from a similar regional practice setting, which may limit generalizability. Future prospective multicenter studies are needed to further validate and refine this model.Future studies may test whether model-guided preparation reduces reduction attempts or operative time and compare this interpretable approach with more complex machine-learning models in independent cohorts (13).

## Data Availability

The raw data supporting the conclusions of this article will be made available by the authors, without undue reservation.
